# Syllable frequency and word frequency effects in spoken and written word production in a non-alphabetic script

**DOI:** 10.3389/fpsyg.2014.00120

**Published:** 2014-02-18

**Authors:** Qingfang Zhang, Cheng Wang

**Affiliations:** ^1^Department of Psychology, Renmin University of ChinaBeijing, China; ^2^Key Laboratory of Behavioral Science, Institute of Psychology, Chinese Academy of SciencesBeijing, China

**Keywords:** spoken production, written production, word frequency effect, syllable frequency effect, Chinese

## Abstract

The effects of word frequency (WF) and syllable frequency (SF) are well-established phenomena in domain such as spoken production in alphabetic languages. Chinese, as a non-alphabetic language, presents unique lexical and phonological properties in speech production. For example, the proximate unit of phonological encoding is syllable in Chinese but segments in Dutch, French or English. The present study investigated the effects of WF and SF, and their interaction in Chinese written and spoken production. Significant facilitatory WF and SF effects were observed in spoken as well as in written production. The SF effect in writing indicated that phonological properties (i.e., syllabic frequency) constrain orthographic output via a lexical route, at least, in Chinese written production. However, the SF effect over repetitions was divergent in both modalities: it was significant in the former two repetitions in spoken whereas it was significant in the second repetition only in written. Due to the fragility of the SF effect in writing, we suggest that the phonological influence in handwritten production is not mandatory and universal, and it is modulated by experimental manipulations. This provides evidence for the orthographic autonomy hypothesis, rather than the phonological mediation hypothesis. The absence of an interaction between WF and SF showed that the SF effect is independent of the WF effect in spoken and written output modalities. The implications of these results on written production models are discussed.

## Introduction

Although effects of word frequency (WF) and syllable frequency (SF) have been investigated systematically in the speech production domain, only a few of studies address similar issue in the written production of normal subjects (i.e., Bonin et al., 1998a; Bonin and Fayol, 2002). As a result, the study of WF and SF is far more advanced in speaking than in writing. The current view of speech production provide a general theoretical framework from which hypotheses specific to writing can be derived. Hence, it could be argued that the generation of written words should be investigated in close parallel to spoken production. In the work reported here, we investigated WF and SF effects, and their interaction in spoken and written production in Chinese, and addressed the general question of whether or not written production is independent or dependent on spoken production.

A central theoretical issue in the field concerns the extent to which written production is autonomous from or dependent on, spoken production. Early theoretical accounts claimed that the retrieval of an orthographic representation was entirely dependent on the prior retrieval of phonological codes, which is called the obligatory phonological mediation hypothesis. Evidence supporting this view comes from the common introspective experience of how written codes are generated (Hotopf, 1980), and the phonologically mediated spelling errors such as homophone substitutions (e.g., there for their) or quasi-homophone substitutions (e.g., dirth for dearth) (Aitchison and Todd, 1982). Neuropsychological patients with writing disorders present comparable impairments in spoken and written language production (Luria, 1970; Basso et al., 1978).

However, other neuropsychological studies have demonstrated dissociations between spoken and written production. For example, Rapp et al. (1997) presented the case of a neurologically impaired individual who was often able to write the names of pictures correctly while being unable to provide the correct spoken names. Miceli et al. (1997) reported a patient who, when presented with a picture, sometimes generated different spoken and written responses (e.g., for picture of *pliers*, he would say *pincers* but write *saw*) (see Alario et al., 2003 for a similar case study). The agraphic patients also produced errors with phonologically illegal spelling (e.g., Caramazza and Miceli, 1990). These findings motivated the “orthographic autonomy hypothesis,” which assumes that individuals can gain access to orthographic representation directly from meaning without phonological mediation (Rapp and Caramazza, 1997).

This account, however, does not necessarily imply that intact writing is unaffected by phonological codes in normal individuals. Relatively few empirical studies have addressed the relationship between phonological and orthographic codes with chronometric tasks, and the results have not been consistent. A few studies have demonstrated that phonological codes indeed influence writing (e.g., Bonin et al., 2001; Zhang and Damian, 2010; Afonso and Álvarez, 2011; Damian et al., 2011). Bonin et al. (2001) manipulated the consistency of phonology-orthography mappings in picture names to identify the potential effects of phonological codes in written picture naming. Word-initial inconsistencies at the sublexical level were found to affect writing latencies: picture names with inconsistent phono-orthographic mapping were written more slowly than those with consistent ones, whereas no difference was found when consistency was manipulated at the lexical level. This finding further suggests that phonology affects orthographic encoding mainly via the sublexical route. In contrast, Bonin et al. (1998b) did not obtain evidence supporting the role of phonology in a picture writing task.

Overall, although some tentative evidence exists suggesting that phonological codes constrain orthographic output tasks such as handwriting, more evidence is needed to resolve this controversial issue. The experiments reported in this article contribute to this debate by comparing the WF effect and the SF effect in spoken and written production. In the following we will sketch a provisional framework which accommodates the effects of WF and SF, and previous studies on the effects of WF and SF.

### The word frequency and syllable frequency effect in speech production

The Word Encoding by Activation and VERification model (WEAVER++) is the most detailed theory about word-form encoding in speech production (Roelofs, 1992, 1997a,b; Levelt et al., 1999). The WEAVER++ assumes two steps for word-form encoding in speech production. A first step is to select the word's form information in the mental lexicon. There are two kinds of word form information: a word's segmental and its metrical form. A morpheme initially activates all its corresponding phonological segments and their order. In parallel to this segmental spell out, metrical codes containing an abstract grouping of syllables into phonological words are retrieved. Both segments and metrical structure (frame) are subsequently merged in a syllabification process in a strictly sequential fashion (segment-to-frame association). The second step is to compute or access the gestural score from the mental syllabary that will result in a phonological word's syllables, and this process is sometimes called phonetic encoding. The mental syllabary can provide pre-compiled gesture scores for phonetic encoding, and the mental syllabary is a store of abstract motor routines of syllabic size. The above-mentioned two steps are successive and independent in the WEAVER++ model.

It has been demonstrated that the retrieval of word form is sensitive to WF as high-frequency words are named faster than low-frequency words in picture naming (Oldfield and Wingfield, 1965; Wingfield, 1968). In Jescheniak and Levelt Study (1994) frequency effects were not obtained in either an object recognition task, or in a delayed word production task. The object recognition task taps into conceptual representations of speech production, while the delayed naming task taps into articulation process, and therefore, the absence of frequency effects in both tasks indicates that the WF effect is lexical in origin. They further demonstrated that the WF effect in word production is due mainly to accessing the phonological forms of words. Other evidence supporting the attribution of frequency effects to phonological forms comes from studies of homophone production (i.e., Stemberger and Macwhitney, 1986; Dell, 1990; La Heij et al., 1999; Jescheniak et al., 2003). However, other researchers (Caramazza et al., 2001; Bonin and Fayol, 2002; Shatzman and Schiller, 2004; Cuetos et al., 2010) failed to find a homophone frequency effect. These findings question the conclusion that the WF effect arises at the phonological forms of a word stage, and supports the lexical origin of WF effect, although they do not deny its influence at the phonological level.

Analogous to the findings for the WF effect, if the mentally syllabary consists of retrievable representations corresponding to syllables, then the stored syllables should exhibit a frequency effect, that is a syllable frequently used in language should be retrieved faster than one less frequently used. Levelt and Wheeldon (1994) tested this storage hypothesis by comparing retrieval latencies for high- vs. low-frequency syllables. They found that words with high frequency syllables were named faster than words with low-frequency syllables when WF was matched. The mental syllabary hypothesis assumes the pre-compiled gestural scores for the articulators rather than constructing the motor programs for each syllable on-line. If a syllable is retrieved from the mental syllabary rather than computed on-line, then the retrieval process should be sensitive to the frequency differences. Therefore, Levelt and Wheeldon interpreted this finding as support for the notion of a mental syllabary.

The hypothesis that content syllables are explicitly represented as chunks and retrieved from a mental syllabary has been tested by investigating whether or not speech production performance is sensitive to SF. SF effects were obtained in a number of studies in different alphabetic languages with words and pseudowords (German: Aichert and Ziegler, 2004; Dutch: Levelt and Wheeldon, 1994; Cholin et al., 2006; Spanish: Perea and Carreiras, 1998; Carreiras and Perea, 2004; French: Laganaro and Alario, 2006; English: Macizo and Van Petten, 2007; Cholin et al., 2011). The effect of SF in spoken output is usually facilitatory, which might be due to faster access to articulatory-phonetic syllable programs for high-frequency syllables (Levelt, 1989; Levelt and Wheeldon, 1994; Levelt et al., 1999). Laganaro and Alario (2006) employed immediate and delayed picture naming and pseudo word naming tasks, with or without articulatory suppression (i.e., repetition of the syllable /ba/) to investigate the assumption that stored syllables are retrieved during phonetic encoding by manipulating syllable frequencies. A SF effect was found in immediate pseudo-word naming, picture naming and in a delayed naming task with articulatory suppression but was not observed in standard delayed naming. As the process of articulatory suppression disrupts phonetic processing but not phonological encoding. This pattern of results is interpreted as evidence that SF affects the phonetic encoding stage.

In sum, available evidence suggests that WF plays a role at the lexical level while SF plays a role during accessing stored syllabic units (a later stage of word-form encoding) in speech production.

### The word frequency and syllable frequency effect in written production

Bonin et al. (1998a,b) have shown that frequency effects in writing are genuinly lexical because they did not find a significant frequency effect either in an object recognition task or in a delayed written picture naming task. Bonin and Fayol (2002) further investigated WF effects in written and spoken production of homophonic picture names, and found homophone frequency effects in spoken as well as in written production: heterographic homophonic picture names with high-frequency were produced faster than low-frequency picture names. They excluded the possibility that the effects arise at the conceptual level in a picture categorization task, and suggested that the WF effect in writing is lexical in origin. According to the obligatory phonological mediation hypothesis, the locus of WF effects should be the same in both spoken and written production, namely at the phonological lexeme level. In contrast, according to the orthographic autonomy hypothesis, which claims that orthographic representations can be accessed directly from semantic representations, the most likely locus of WF effects is at the orthographic lexeme level.

We are not aware of any studies examining the SF effect in written production. However, a few studies have demonstrated that syllables modulate processes of written production. Kandel et al. (2006a,b) observed that French 1st–5th graders write words and pseudowords syllable by syllable, reflecting that the children used the syllable as a unit for chunking letter strings in a coherent way. The syllable effect in handwriting has been demonstrated in other developmental studies (Kandel et al., 2006b) as well as in adults (Kandel et al., 2006a; Lambert et al., 2007). Kandel and her colleagues examined the nature of syllabic processing in children (Kandel et al., 2009). They manipulated orthographic and phonological matched or mismatched syllables in French word writing task. Third, 4th, and 5th graders were asked to write words that were mono-syllables phonologically (i.e., barque is [baRk]) but bi-syllables orthographically (i.e., barque = bar.que), which matched to words that were bi-syllables phonologically and orthographically (i.e., balcon = [bal.kõ] and bal.con). They found that results on letter stroke duration and fluency generated significant peaks at the syllable boundary for both types of words, reflecting that children use orthographic syllables rather than phonological syllables in handwriting production.

So far, word and syllable frequency effects have to our knowledge not been investigated in Mandarin Chinese, a non-alphabetic language. In alphabetic languages such as Spanish, syllables are predictable from orthography, and Dutch, French or English syllables are also predictable, are although less so than Spanish. By contrast, it is totally unpredictable in Chinese. Studies indicated that it is possible that a syllabic effect is not a phonological effect but an orthographic syllabic effect in written production (see also Kandel et al., 2009). Due to the unique characteristics of Chinese, a study of the SF effect in Chinese would clarify the role of phonology in written production.

A few studies addressed the role of the syllable in Chinese spoken production. O'Seaghdha et al. (2010) proposed a model of sequential steps in word form encoding in Mandarin Chinese monosyllabic word production. Similar to WEAVER++, content and structure are separated in the model. Activation from the corresponding abstract word flows to phonological content and structure. Phonological content is activated as syllables, while their syllabic frames are retrieved. Both syllabic content and frame are linked sequentially and metrical tone is also specified at this point. In contrast to WEAVER++ model of alphabetic languages, syllables are chunks in Mandarin Chinese.

According to O'Seaghdha et al. (2010), syllables are proximate units and are retrieved from the mental lexicon at an early stage of phonological encoding. This has been supported by several studies. For example, there are many syllable-sized phonological speech errors in Chinese, whereas segmental errors are quite rare (Chen, 1993, 2000). Chen et al. (2003) investigated the role of the syllable using a masked priming task as Ferrand and his colleagues had employed in French (Ferrand et al., 1996, 1997). Disyllabic Mandarin Chinese words were used as targets and single Chinese characters were used as primes. In Chen et al's third experiment, syllable overlap between prime and the first syllable of a disyllabic target was manipulated. They found that the CV targets were named faster when preceded by CV primes compared to the CVG (G represents glide sound) primes, whereas the opposite pattern was obtained for the CVG targets. The critical crossover interaction between prime type and target type was significant, and thus provides evidence for the notion that the syllable is a functional unit in speech production. You et al. (2012) obtained syllable priming effects across different stimuli and different tasks (word and picture naming), and provide more conclusive data regarding the role of the syllable in Chinese spoken production. In addition, studies using other production tasks such as the implicit priming task (Chen et al., 2002; O'Seaghdha et al., 2010) and the picture-word interference task in spoken production (Zhang, 2008; Zhang and Weekes, 2009; Zhang et al., 2009) and in written production (Qu et al., 2011) also attested to the important role of the syllable in Chinese. In contrast, most studies in Dutch, French and English demonstrated that the proximate unit is segments in alphabetic languages (Schiller, 1998, 1999, 2000; Brand et al., 2003). These contrastive findings suggest that the role of the syllable in Chinese is different from Dutch, English or French.

On the other hand, to the best of our knowledge, only a few experimental studies involving normal participants have investigated the extent to which the processes and the representations involved in speech production resemble those involved in written production (Bonin et al., 1997, 1998a,b; Bonin and Fayol, 2000) and then only in French.

In the present study, we aim to investigate the effects of WF, SF and their interaction, on spoken and written production latencies. Although spoken and written language production systems obviously share some processing levels, they also both have some specific processing components (Bonin et al., 1998a). Picture naming and writing are thought to differ beyond the conceptual-semantic level: a phonological lexeme level in naming and an orthographic lexeme level in writing (Ellis, 1982, 1988; Caramazza and Hillis, 1990). Phonological information can serve as input for articulation in spoken production and orthographic information can serve as input for orthographic output in written production. Writing also involves the retrieval of an orthographic plan as well as the execution of a motor program, which is different from speaking execution.

Given that the WF and SF effects are well-established phenomena in domains such as spoken production, it is plausible to predict similar effects in orthographic output tasks in the framework of the obligatory phonology mediation hypothesis, because the retrieval of orthographic codes depends on the retrieval of phonological codes. Our basic assumption was that, similar effects would indicate that similar processes are involved in both forms of language production: A SF effect in Chinese written production would provide support for the phonological mediation hypothesis due to deep mapping of orthography-to-phonology in Chinese. In contrast, in the framework of the orthographic autonomy hypothesis, the retrieval of orthographic codes does not require access to phonological codes, and orthographic and phonological representations can be accessed independently. We predict WF effects would differ in spoken and written output, and the absence of SF effect in written output.

A second purpose of the experiment was to assess the robustness of the effects over repetitions. Are the word and SF effects ephermeral, that is, do they dissipating with repeated use of the word? Or is it structural, insensitive to repeated processing of an individual item (see also Jescheniak and Levelt, 1994).

## Experiment 1: word frequency and syllable frequency in speaking

### Methods

#### Participants

Twenty-four students (12 males, average 23.2 years, range 20–25 years) participated and were paid approximately $3. They were randomly taken from Beijing Forest University and China Agricultural University. All were native Mandarin Chinese speakers with normal or corrected-to-normal vision.

#### Materials

Sixty target pictures with monosyllabic names were selected from Zhang and Yang's (2003) picture database. Word frequencies were taken from the Modern Chinese Frequency Dictionary (Beijing Language Institute, 1986). The mean number of strokes of target names is 9.95. A Chinese character's pronunciation (pinyin) corresponds to one syllable, and thus SF was calculated by accumulating the word frequencies of one syllable (not counting tone). For 60 monosyllabic words, half were high frequency (all ≥130/per million), half were low frequency (≤47/per million). Among high and low frequency words, half had high SF (≥2558/per million), half had low SF (≤1479/per million). Note that, low-frequency syllables had above-average frequency of occurrence in the language. This is important as the WEAVER++ model claims that very low-frequency syllables will be formed on-line rather than retrieved from the mental syllabary. Thus, we used relatively low-frequency syllables in the experiment. Table 1 shows the properties of picture names and pictures used in the experiments. Statistical analyses showed that a significant difference between low and high WF [*t*_(58)_ = 9.42, *p* < 0.0001], and a significant difference between low and high SF [*t*_(58)_ = 9.57, *p* < 0.0001). Statistical analyses showed no significant difference between low and high WF on naming consistency, familiarity, image consistency, and complexity. All items are reported in Appendix.

**Table 1:** **Means word frequency (per million), syllable frequency (per million), number of neighbors, strokes number, naming consistency, familiarity, image consistency, and image complexity of the stimuli**.

**Types**	**Word frequency**	**Syllable frequency**	**Number of neighbors**	**Strokes number**	**Naming consistency**	**Familiarity**	**Image consistency**	**Image complexity**
**HIGH FREQUENCY WORDS**								
High frequency syllables	297	5898	9.27	9.13	1.06	4.80	3.76	2.42
Low frequency syllables	255	869	2.87	9.13	1.24	4.32	3.19	2.50
**LOW FREQUENCY WORDS**								
High frequency syllables	28	5253	8.40	10.87	1.05	4.29	3.46	2.34
Low frequency syllables	22	716	3.47	10.67	1.05	4.39	3.71	2.51

#### Design

The experimental design included (WF: low vs. high) and SF (low vs. high) and Repetition (1st, 2nd, and 3rd) as within-participants factors. Each participant names 60 target words three times, resulting in 180 trials in total. Each repetition was set in one block, and thus there were three blocks in total. The order of target words within a block was pseudo-randomized to prevent targets with the same onset repeating across five trials. A new sequence was generated for each participant and each block.

#### Apparatus

The experiment was performed using E-Prime Professional Software (Version 1.1; Psychology Software Tools). Participants were seated in a quiet room approximately 70 cm from a 19 inch LED computer screen. Naming latencies were measured from target onset using a voice-key, connected to the computer via a PST Serial Response Box.

#### Procedure

Participants were tested individually. They sat in a dimly lit room at a comfortable viewing distance in front of the computer. Before the experiment, participants were instructed that their task was to name pictures. Participants first were asked to familiarize themselves with the experimental stimuli by viewing each target for 3000 ms with the correct name printed below. Then, 4 warm-up trials and 60 trials for each repetition were administered.

Participants were asked to name pictures as quickly and accurately as possible. Each trial involved the following sequence: A fixation point (+) presented in the center of the screen for 500 ms, followed by a blank screen for 500 ms. After that the target picture appeared, an inter-trial interval of 1500 ms concluded each trial. The experiment took about 30 min in total.

#### Results

**Figure 1:**
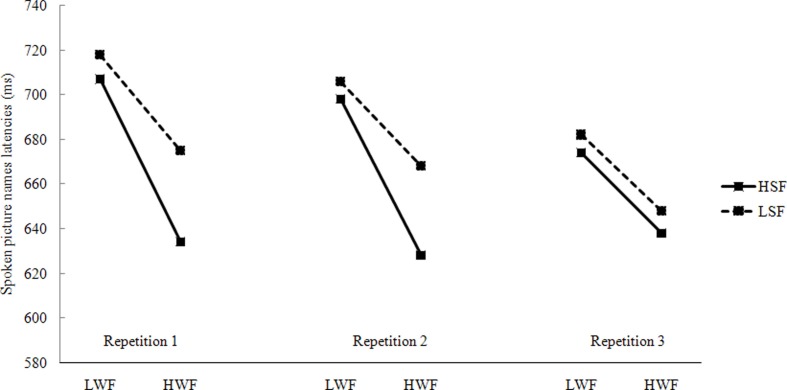
**Mean naming latencies in spoken responses by WF, SF, and repetitions (L, Low; H, High; WF, Word Frequency; SF, Syllable Frequency)**.

Data from incorrect responses (0.39%), naming latencies longer than 1500 ms or shorter than 200 ms (0.58%), and latencies deviating 2.5 standard deviations from the cell mean (2.27%) were removed from all analyses. The remaining data were used in the subsequenct statistical analysis. Figure 1 presents the mean latencies, presented by Character Frequency, SF, and Repetition in spoken picture naming.

We used the lmer program of the lme4 package for estimated fixed effects and parameter estimation of the LMM (Bates, 2005; Baayen et al., 2008; Bates et al., 2009). The free software R was used (R Development Core Team, 2009). The data were analyzed using a linear mixed-effects model that included fixed effects of WF, SF, and Repetition, and by-participant and by-item random intercepts. Models were fit to the data using a restricted maximum likelihood estimation, which seeks to find those parameter values that make the model's predicted values most similar to the observed values. Model fitting was carried out by initially specifying a model that only included the random factors (participants and items) which was then enriched by subsequently adding the fixed factors WF, SF, and Repetition one by one, followed by the interaction between WF and Repetition, the interaction between WF and SF, the interaction of SF and repetition, and the 3-way interaction among WF, SF, and Repetition one by one. The best fitting model was defined to be the most complex model that significantly improved the fit over the previous model. If adding a fixed factor or an interaction among factors did not significantly improve the fit, this indicates that they do not produce significant influences on the dependent variables (i.e., naming latencies).

For speaking latencies, the best fitting model included WF, SF, Repetition, the interaction between WF and Repetition (see Table 2). Adding the interactions between WF and SF, χ^2^_(1, 4180)_ = 0.61, *p* = 0.43, SF and Repetition, χ^2^_(2, 4180)_ = 4.67, *p* = 0.10, and the triple interaction among WF, SF, and Repetition, χ^2^_(2, 4180)_ = 3.41, *p* = 0.18, did not significantly improve the fit. Data analysis indicated a significant WF effect and a marginally significant SF effect across repetitions. In order to examine the effects of WF and SF, and their interaction, speaking data were analyzed separately for each repetition.

**Table 2:** **LMM estimates of fixed effects for latencies in speaking**.

**Fixed effects**	**Measure**		
	**Estimate**	**Std. Error**	***t*-value**
(Intercept)	725.33	18.37	39.48
WF2	−59.80	13.32	−4.49^****^
SF2	−19.64	12.42	−1.58^£^
rep2	−11.94	5.89	−2.03^**^
rep3	−34.70	5.91	−5.87^****^
WF2:rep2	5.64	8.32	0.68
WF2:rep3	23.87	8.34	2.86^***^

WF2, High word frequency; SF2, High syllable frequency.

£ *p < 0.10*,

** *p < 0.05*,

*** *p < 0.01*,

**** p < 0.001.

For the first and the second repetitions, the best fitting model included WF and SF, adding the interaction between WF and SF did not significant improve the fit in the first repetition, χ^2^_(1, 1396)_ = 0.86, *p* = 0.35, and the second repetition, χ^2^_(1, 1397)_ = 1.26, *p* = 0.26. For the third repetition, the best fitting model included WF only, adding the SF, χ^2^_(1, 1387)_ = 0.60, *p* = 0.44, and the interaction between WF and SF, χ^2^_(1, 1387)_ = 0.00, *p* = 1, did not significantly improve the fit. Table 3 displays parameter estimates for fixed effects in each repetition.

**Table 3:** **LMM estimates of fixed effects for latencies for each repetition in speaking**.

**Effect**	**Measure**								
	**The 1st repetition**			**The 2nd repetition**			**The 3rd repetition**		
	**Estim**	***SE***	***t***	**Estim**	***SE***	***t***	**Estim**	***SE***	***t***
(Intercept)	728.45	20.14	36.18	715.32	18.37	38.94	680.05	17.24	39.45
WF2	−60.24	14.92	−4.04^****^	−54.28	13.54	−4.01^****^	−35.42	11.12	−3.19^***^
SF2	−25.71	14.92	−1.72^*^	−23.70	13.54	−1.75^*^	–	–	–

WF2, High word frequency; SF2, High syllable frequency.

* *p < 0.08*,

*** *p < 0.01*,

**** p < 0.001.

A parallel analysis was conducted on the errors, but a binomial family was used because of the binary nature of the responses. For each repetition, no models including WF, SF, or their interaction significantly improve the fit, χ^2^_(1)_s ≤ 1.29, *p* ≥ 0.26. Planned comparisons showed no significant effects of WF, *zs* ≤ 0.28, *ps* ≥ 0.75, and no significant effects of SF, *zs* ≤ 0.74, *ps* ≥ 0.46.

#### Discussion

The experimental results are clear-cut. First, a highly reliable WF effect (average: 49 ms) was obtained, pictures with high-frequency names were produced faster and more accurately than those with low-frequency names. Although the WF effect decreased from the first (58 ms) repetition to the third (35 ms) repetition, it was still a substantial 35 ms in the third repetition. This finding is consistent with other studies that showed that the WF effect decreases with repeated presentations of the same set of pictures (e.g., Bartram, 1973; Monsell et al., 1992; Wheeldon and Monsell, 1992; Griffin and Bock, 1998).

Second, the experiment showed that there is a SF effect in the first and the second repetitions, pictures with high-frequency syllables produced faster than those with low-frequency syllables. This was in accordance with previous findings (Levelt and Wheeldon, 1994). According to the WEAVER++ model, syllables are retrieved from the mental syllabary, therefore, a SF effect was observed. However, this effect dissipated in the third repetition. What could be the cause of the ephemeral SF effect? We suggest that it is a recency effect (see also Jescheniak and Levelt, 1994) for a similar pattern on a gender decision task]. After two repetitions, high- and low- frequency syllables became equated on recency, and thus the SF effect decreased or disappeared in the third repetition.

Third, there was no interaction between WF and SF in spoken picture naming, indicating that the SF effect is independent of the WF effect (see also Levelt and Wheeldon, 1994 for a similar conclusion).

## Experiment 2: word frequency and syllable frequency in writing

### Methods

#### Participants

Twenty-four students (11 males, average 23.0 years, range 19–28 years) from the same student pool participated in the experiment and were paid approximately $3. None of them participated in Experiment 1.

#### Materials and design

They were identical to experiment 1.

#### Apparatus

The experiment was run using the E-Prime Professional Software (Version 1.1; Psychology Software Tools). The computer controlled the presentation of the pictures and recorded the latencies. Written responses (the intervals between picture onset and initial contact of the pen on the writing surface) were recorded via a WACOM Intuos A4 graphic tablet and a WACOM inking digitizer pen (WACOM, Japan) connected to the computer. Other details were identical to experiment 1.

#### Procedure

Pictures were displayed at the bottom of the screen in order to reduce participants' head and eye movements as they wrote the picture names. Participants were asked to write picture names as quickly and accurately as possible. During the experiment, participants were instructed to hover the stylus just above the corresponding line on the sheet in anticipation of the response, so that the response would not require an arm movement.

Each trial involved the following sequence: A fixation point (+) presented at the bottom of the screen for 500 ms, followed by a blank screen for 500 ms. After that the target picture appeared, an inter-trial interval of 3500 ms concluded each trial. The experiment took about 40 min in total. Other procedures were identical to experiment 1.

#### Results

**Figure 2:**
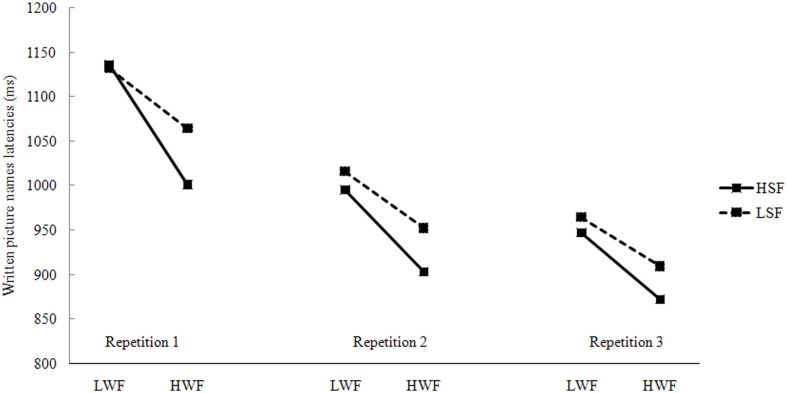
**Mean naming latencies in written responses by WF, SF, and repetitions (L, Low; H, High; WF, Word Frequency; SF, Syllable Frequency)**.

Data from incorrect responses (0.81%), writing latencies longer than 2000 ms or shorter than 300 ms (1.88%), and latencies deviating 2.5 standard deviations from the cell mean (2.36%) were removed from all analyses. The remaining data were used in the subsequent statistical analysis. Figure 2 presents the mean written latencies, presented by WF, SF, and Repetition.

The data were analyzed using a linear mixed-effects model that included fixed effects of WF, SF, and Repetition, and by-participant and by-item random intercepts. Results are reported for the best-fitting models.

For writing latencies, the best fitting model included WF, SF, Repetition, and the interaction between WF and Repetition (see Table 4). Adding the interactions between WF and SF, χ^2^_(1, 4102)_ = 0.90, *p* = 0.34, SF and Repetition, χ^2^_(1, 4102)_ = 0.35, *p* = 0.84,, and the triple interaction among WF, SF, and Repetition, χ^2^_(2, 4102)_ = 3.42, *p* = 0.18, did not significantly improve the fit. Data analysis indicated a significant WF effect and a marginally significant SF effect across repetitions. In order to examine the effects of WF and SF, and their interaction, writing data were analyzed separately for each repetition.

**Table 4:** **LMM estimates of fixed effects for latencies in writing**.

**Fixed effects**	**Measure**		
	**Estimate**	**Std. Error**	***t*-value**
(Intercept)	1154.14	27.90	41.36
WF2	−104.19	19.07	−5.46^****^
SF2	−30.37	17.30	−1.76^*^
rep2	−130.04	9.75	−13.34^****^
rep3	−182.86	9.73	−18.80^****^
WF2:rep2	24.94	13.72	1.82^*^
WF2:rep3	39.95	13.70	2.92^**^

WF2, High word frequency; SF2, High syllable frequency.

* *p < 0.08*,

** *p < 0.05*,

**** p < 0.001.

For the first and the third repetitions, the best fitting model included WF only. In the first repetition, adding SF, χ^2^_(1, 1335)_ = 1.58, *p* = 0.21, and the interaction between WF and SF, χ^2^_(1, 1335)_ = 1.88, *p* = 0.17, did not significant improve the fit, In the third repetition, adding SF, χ^2^_(1, 1389)_ = 2.49, *p* = 0.11, and the interaction between WF and SF, χ^2^_(1, 1389)_ = 0.26, *p* = 0.61, did not significant improve the fit. For the second repetition, the best fitting model included WF and SF, adding the interaction between WF and SF, did not significantly improve the fit, χ^2^_(1, 1378)_ = 0.36, *p* = 0.55. Table 5 displays parameter estimates for fixed effects in each repetition.

**Table 5:** **LMM estimates of fixed effects for latencies for each repetition in writing**.

**Effect**	**Measure**								
	**The 1st repetition**			**The 2nd repetition**			**The 3rd repetition**		
	**Estim**	***SE***	***t***	**Estim**	***SE***	***t***	**Estim**	***SE***	***t***
(Intercept)	1138.46	30.28	37.60	1026.65	28.37	36.19	955.60	25.27	37.81
WF2	−103.22	22.81	−4.53^****^	−80.08	17.28	−4.63^****^	−64.37	16.88	−3.81^****^
SF2	–	–	–	−35.29	17.28	−2.04^**^	–	–	–

WF2, High word frequency; SF2, High syllable frequency.

** *p < 0.05*,

**** p < 0.001.

A parallel analysis was conducted on the errors, but a binomial family was used because of the binary nature of the responses. For each repetition, no models including WF, SF, or their interaction significantly improve the fit, χ^2^_(1)_s ≤ 2.43, *p* ≥ 0.12. Planned comparisons showed no significant effects of WF, *zs* ≤ 1.03, *ps* ≥ 0.30, and no significant effects of SF, *zs* ≤ 1.43, *ps* ≥ 0.15.

#### Discussion

Similar to spoken naming, we observed a highly reliable WF effect (average: 81 ms), which was larger than the effect in spoken naming. The WF effect decreased obviously but was still significant in the third repetition. The most obvious explanation is that it reflects the participants' accommodation to the task (see also Jescheniak and Levelt, 1994). Significant SF effect was only observed in the second repetition only. There was no interaction between WF and SF in written naming as well.

## General discussion

We employed spoken naming and written naming tasks to investigate effects of WF and SF, and their interaction. The main findings of the two experiments are these: (1) SF and WF affect naming and writing latencies; (2) the SF effect is independent of WF in both output modalities; (3) the WF effect is attenuated with the repetition of the pictures but still persisted even after three repetitions in both output modalities. The magnitude of WF effect was larger in written output than in spoken output. (4) the SF effect disappeared in spoken output after two repetitions, while it was there in written response in the second repetition only. This indicates that the phonological influence in handwritten production is not mandatory and universal, and it was modulated by experimental manipulations.

In non-alphabetic languages such as Chinese, we obtained WF and SF effects in spoken production. Pictures with high WF names were produced 56 ms faster than those with low WF names, an effect size similar to Jescheniak and Levelt's findings (i.e., 62 ms) in picture naming in Dutch. Additionally, the WF effect was invariant over the first two repetitions, and decreased in the third repetition but was still significant. This indicates that the WF effect is not easily influenced by repetitions (see Jescheniak and Levelt, 1994 for a similar finding). Given the assumption that naming pictures involves identification, conceptual preparation, lexicalization, and output preparation, and that the pictures were matched on naming consistency, familiarity, image consistency, and imaging complexity (see Table 1), in combination with evidence from previous studies with the delayed naming task (Jescheniak and Levelt, 1994; Almeida et al., 2007), we suggest that the WF effect might be lexical in origin in Chinese spoken production, although we cannot determine its exact locus (either an early stage i.e., lemma selection or a later stage i.e., word-form encoding) based on our findings.

In alphabetic languages, notably Dutch, it has been demonstrated that SF effect arise at the stage of phonetic encoding, and may relate to faster access to a mental syllablary, where syllables are stored separately from words (Levelt and Wheeldon, 1994; Levelt et al., 1999; Aichert and Ziegler, 2004; Carreiras and Perea, 2004; Cholin et al., 2006, 2011; Laganaro and Alario, 2006). These findings come largely from alphabetic languages such as Dutch, German, French, and English. Studies in Chinese as a non-alphabetic language also suggest that the syllable plays a prominent role (Chen et al., 2002; You et al., 2012). Furthermore, O'Seaghdha et al. (2010) propose a different position for the stored syllabic units in Chinese. They assume that there are proximate units which are the first selectable phonological units below the level of the word/morpheme and vary across languages. The proximate units are syllables in Mandarin Chinese (Chen et al., 2002; You et al., 2012; but see Wong and Chen, 2008, 2009) but segments in alphabetic languages (Schiller, 1998, 1999, 2000, 2008). According to the assumption of proximate units, in Chinese, phonological content is bundled in syllables with links it to the corresponding unit in the structure network and a syllable frame. Then, the segments of a syllable are linked to positions in the syllable frame. We therefore suggest that the SF effect originates at an early stage of word-form encoding in spoken responses in Chinese, rather than at the stage of phonetic encoding. This is the first study reporting a SF effect in Mandarin Chinese a non-alphabetic language. From the present results we conclude that Chinese is sensitive to SF manipulations in speaking, although its locus might be different from alphabetic languages.

As far as written production is concerned, we observed a SF effect in written responses in the second repetition only, suggesting that phonological codes may constrain the generation of orthographic output codes. However, the SF effect was not observed in the first and the third repetitions. This indicates that the effect of phonological codes in handwritten production was modulated by the manipulation of repetitions. Zhang and Damian (2010) found that the phonological facilitation effect disappeared when an articulation suppression task was performed during the preparation of written responses, reflecting that the phonological influence is not mandatory and universal, and it can be modulated via the manipulation by an articulation suppression task (see also Shen et al., 2013 for a similar conclusion). On the other hand, a large size of WF effect was observed in writing and it rapidly decreased over repetitions but still significant in the third repetition.

Concerning the SF effect, it was significant in the first and the second repetitions but not in the third repetition in speaking. Jescheniak and Levelt (1994) also observed that the WF effect was robust over repetitions in picture naming, but it rapidly decreased and disappeared by the third repetition in a gender decision task. They suggested that this reflects a recency effect: high- and low- WF became equated on recency after two repetitions, thus the WF effect decreased or disappeared in the third repetition. In contrast, we did not observe this pattern in written output. Why does the recency effect arise in spoken responses but not in written responses? Accessing phonological codes is mandatory in spoken but not in written (Zhang and Damian, 2010; Shen et al., 2013), and participants need to encode phonological information for articulation in speaking. In other words, the process of speaking is sensitive to SF. In contrast, it is not necessary to access the phonological codes in writing and thus the process of writing is insensitive to SF. However, it should be note that the magnitude of the SF effect was comparable in spoken (average 25 ms in the first and the second repetitions) and written responses (average 27 ms in three repetitions). This reflected that written production involve similar syllabic processing to spoken production in Chinese. This relatively weak effect cannot imply that syllable does not play a role in speaking and writing, because large numbers of studies demonstrated that the role of syllable in spoken production (e.g., Levelt and Wheeldon, 1994; You et al., 2012). A possibility was that writing latencies were much longer than speaking latencies (991 ms vs. 673 ms), therefore, a 27-ms SF effect was not significant in longer latencies. Chen and Cherng (2013) found that reaction times were around 680 ms from the onset of a cue character to the onset of a written response with a prompt-response generation task. Therefore, further study could use this more sensitive task to investigate the SF effect in written production.

A larger WF effect was observed in written (average: 81 ms) than in spoken (average: 49 ms), suggesting that written production may involve different processes from spoken production. The written model proposed by Bonin et al. (2001) assumes a semantic system that is symmetrically linked to both a phonological and an orthographic output lexicon. Both lexicons also directly map onto each other, implying that selection of a graphemic entry is influenced by both direct activation from the semantic system and indirect activation from the phonological lexicon. The model also assumes a sublexical phonology-to-orthography conversion route, paralleling the sublexical grapheme-to-phoneme route in dual-route models of reading aloud (Coltheart et al., 2001). From the SF effect, we infer that phonological lexeme might contribute to the WF effect in written as well as in spoken production. On the other hand, orthographic representations might be accessed directly from semantic representations. The locus of WF effect would then originate from the orthographic lexemes level (see also Bonin and Fayol, 2002). Hence, there are two possible resources for the WF effect: one is at the orthographic lexeme level, and the other is at the phonological lexeme level.

The latencies were systematically longer in written than in spoken production in the present study, therefore, differences in the WF effect between written and spoken was possibly the result of post-lexical processing (see also Bonin et al., 1998a,b; Perret and Laganaro, 2013) or other characteristics specific to handwriting such as monitoring visual control (Smyth and Silvers, 1987; Graham and Weintraub, 1996; Bonin et al., 1998a,b; Alamargot et al., 2006). Bonin et al. (1998a) suggested that the increased latencies did not arise during central cognitive processes, because this difference in latencies between written and spoken is also observed in a delay production task. In contrast, Perret and Laganaro (2013) demonstrated that only when participants can see and monitor their written output, latencies are longer for writing than for speaking. They suggested that this difference is not due to central cognitive processes or a characteristic of post-lexical processes, but the additional actions such as eye movements or control of handwriting's onset. According to these findings, the larger WF effect in written than in spoken production might originate from multiple levels, and needs to be investigated further.

In summary, we firstly observed a WF effect and a SF effect in spoken as well as in written responses in Chinese, and the SF effect is independent of the WF effect. Due to the fragility of the SF effect in writing, we suggest that the phonological influence in handwritten production is not mandatory and universal, and it is modulated by experimental manipulations. This provides evidence for the orthographic autonomy hypothesis, rather than the phonological mediation hypothesis. The findings reported in the article allow only limited insight into the exact nature of phonological codes in written production in Chinese. It is obvious that more intensive research is needed to investigate the impact of WF and SF, and their effects on spoken and written production in Chinese.

### Conflict of interest statement

The authors declare that the research was conducted in the absence of any commercial or financial relationships that could be construed as a potential conflict of interest.
